# Transdiagnostic mechanisms of mental health during the COVID-19 pandemic: associations of childhood trauma, maladaptive personality traits, emotion regulation, mentalizing, and pandemic-related distress

**DOI:** 10.3389/fpsyg.2024.1427469

**Published:** 2024-12-18

**Authors:** Julia Holl, Anna Berning, Laura Kling, Svenja Taubner, Anna K. Georg, Jana Volkert

**Affiliations:** ^1^Department of Clinical Psychology and Psychotherapy, Psychological Institute, University Heidelberg, Heidelberg, Germany; ^2^Institute for Psychosocial Prevention, Centre for Psychosocial Medicine, University Hospital Heidelberg, Heidelberg, Germany; ^3^Chair of Translational Psychotherapy Research, Clinic for Psychosomatic Medicine and Psychotherapy, University Ulm, Ulm, Germany

**Keywords:** Corona virus, child maltreatment, emotion dysregulation, mentalization, depression, anxiety, structural equation modeling

## Abstract

**Introduction:**

The outbreak of the COVID-19 pandemic has led to increased psychological distress. Transdiagnostic factors, including childhood trauma, maladaptive personality traits (MPTs), mentalizing, and emotion dysregulation are considered relevant to the development and maintenance of mental health problems. These factors probably play a significant role in individuals’ reactions to pandemic-related distress (PR distress). The aim of this study is to examine the associations with these transdiagnostic factors in relation to PR distress and psychopathological symptoms.

**Materials and methods:**

Using a cross-sectional study design, we investigated a convenience sample of 6,451 adults (mean age = 44.1; *SD* = 11.8; 69.1% female, 30.3% male, 0.01% diverse) recruited via social media platforms in German-speaking countries (August 2020 – February 2021) by utilizing self-report instruments (PID5BF+, MentS, DERS-SF, PHQ-9, GAD-7, a composite PR distress score). Structural equation modeling was performed for data analysis.

**Results:**

The results revealed significant associations between different types of childhood trauma and MPTs (−0.14 < β < 0.48) as well as a parallel mediation of the relationship between MPTs and psychopathological symptoms via mentalizing (β_anxiety_ = −0.03; β_depression_ = 0.01) and emotion dysregulation (β_anxiety_ = 0.24; β_depression_ = 0.23).

**Discussion:**

Mentalizing and emotion dysregulation seem to play a significant role in relation between childhood trauma and MPTs and psychopathological symptoms during the COVID-19 pandemic. Thus, transdiagnostic factors may be a valuable target for the development of interventions aiming to reduce psychological distress related to a pandemic or other crises events. Specific prevention and intervention methods that target emotion dysregulation and mentalizing could help vulnerable individuals, particularly those with childhood trauma and MPTs, to protect against or alleviate the detrimental effects of PR distress on their mental health.

## 1 Introduction

After the outbreak of the COVID-19 pandemic, the prevalence rates of psychopathological symptoms and disorders substantially increased worldwide (Santomauro et al., 2021). Findings from longitudinal studies and meta-analyses showed increases in depression and anxiety, particularly relative to pre-pandemic levels (Robinson et al., 2022). The majority of studies that focused on mental health distress during the COVID-19 pandemic used measures of general psychopathology, which, as such, do not specifically consider psychological adjustment problems or pandemic-related distress (PR distress). However, several influential conceptual models consider the outbreak of the COVID-19 pandemic as a specific stressor related to psychopathology (McLaughlin et al., 2022). In this respect, the *stress sensitization model* can serve as a framework to examine the development and maintenance of psychological distress and the increase of psychopathological symptoms during the COVID-19 pandemic. The model postulates that experiencing early-life adversity increases the vulnerability for developing psychopathology in response to stressors later in life (Hammen et al., 2000). In line with this view, a large body of empirical studies showed that childhood trauma (i.e., emotional, physical, and sexual abuse as well as emotional and physical neglect) is one of the most important transdiagnostic vulnerability factors for psychopathology (Hogg et al., 2022). Specifically, both cross-sectional (Janiri et al., 2021) and prospective findings (Seitz et al., 2021) indicate that childhood trauma is associated with increased vulnerability to the stressful effect of the pandemic and with increased psychopathology. Accordingly, experiences of childhood trauma can be understood as a vulnerability factor for psychopathology, also in the context of the COVID-19 pandemic. Moreover, specific forms of childhood trauma are considered to have differential effects on a psychopathological development (Infurna et al., 2016). Accordingly, Janiri et al. (2021) found that, in particular, experiences of emotional abuse were associated with increased psychological distress during the COVID-19 pandemic. However, only few studies have investigated the differential effects of specific forms of childhood trauma on psychopathological symptoms during the COVID-19 pandemic.

Within the stress sensitization model, McLaughlin et al. (2020) proposed that childhood trauma is linked to psychopathology through transdiagnostic mechanisms including changes in *emotional processing*, *social information processing*, and *accelerated biological aging*. Emotional processing draws on the concept of emotion regulation, which is well-established in clinical research, for example, due to consistent findings that trauma-exposed individuals experience difficulties with regulating their emotions (McLaughlin et al., 2020). Social information processing (i.e., the perception, identification, and interpretation of social cues; McLaughlin et al., 2020) includes aspects captured by the concepts of personality (Bender et al., 2011) and mentalizing (Luyten et al., 2020). Considering the role of childhood trauma regarding PR distress (Janiri et al., 2021), these transdiagnostic factors may also play a significant role in individuals’ reactions to the pandemic.

In a related line of research, childhood trauma has been highlighted as a factor contributing to the development of personality psychopathology. A recent review indicated significant associations between physical, emotional, and sexual abuse and personality functioning and maladaptive personality traits (MPTs), based on the new dimensional personality model of the DSM-5 and ICD-11 (Back et al., 2021). In this model, MPTs are based on the established Big-Five model, including the factors negative affectivity, detachment, antagonism, disinhibition, and psychoticism (American Psychiatric Association, 2022; Zimmermann et al., 2020). MPTs show strong associations with a variety of mental disorders (Kernberg and Caligor, 2005; Bender et al., 2011). With regard to the pandemic, internalizing MPTs (i.e., detachment, negative affect, psychoticism) have been identified as risk factors for depression, anxiety, and stress symptoms (Mazza et al., 2020; Biondi et al., 2021).

The ability to mentalize is defined as the basic human capacity to understand human behavior in terms of mental states such as feelings, thoughts, desires, attitudes, and goals (Fonagy et al., 2002), whereas impairments in mentalizing are associated with several mental illnesses (Luyten et al., 2020). In the stress sensitization model, social information processing capacities (e.g., mentalizing) are regarded as a mediator between childhood trauma and psychopathology (McLaughlin et al., 2020). Based on the association of impaired mentalizing with mental health problems, considering the context of PR distress, several mentalization-based psychosocial interventions have been developed in response to the pandemic (Ventura Wurman et al., 2021). Only a few empirical studies that have investigated pandemic-related effects on mentalizing found a decrease in parental mentalizing (Yatziv et al., 2022) and an association between psychopathological symptoms and decreased mentalizing (Charpentier Mora et al., 2022). However, none of these studies focused on mentalizing as a mediating risk or protective factor in the general population during the COVID-19 pandemic.

With regard to the emotional processing component in the stress sensitization model, poor emotion regulation is also regarded as a mediator between childhood trauma and psychopathology (McLaughlin et al., 2020). Gratz’s and Roemer (2004) offer a multi-faceted conceptualization of emotion dysregulation as comprising “(a) awareness and understanding of emotions, (b) acceptance of emotions, (c) ability to control impulsive behaviors and behave in accordance with desired goals when experiencing negative emotions, and (d) ability to use situationally appropriate emotion regulation strategies flexibly to modulate emotional responses as desired in order to meet individual goals and situational demands” (Gratz and Roemer, 2004, p. 42). Emotion dysregulation has been identified as a mediator in the relationship between MPTs and psychopathology (Abdi and Pak, 2019). In addition to this, a recent study found that a COVID-19 infection was associated with a greater likelihood of psychological distress, and in turn was associated with greater emotion dysregulation as well as elevated levels of depressive mood (Janiri et al., 2021). Furthermore, current findings also indicate positive associations between mentalizing and emotion dysregulation (Ciccarelli et al., 2021). However, the relationship within the context of the pandemic is still unclear.

Taken together, previous studies on psychopathological symptoms during the pandemic focused on prevalence rates of these symptoms (e.g., Santomauro et al., 2021; Holman et al., 2020) as well as the identification of pandemic-related stressors and their associations with psychopathological symptoms (e.g., Fisher et al., 2022; Shapiro et al., 2020). The objective of this study is to address the existing research gap by investigating key psychological risk and protective factors for adaptive functioning during the COVID-19 pandemic in a nonclinical sample. Based on the stress sensitization model, the overarching aim was to investigate the relative importance of these different factors in predicting psychopathology during the COVID-19 pandemic within one comprehensive model. Particularly, we aimed to investigate how childhood trauma, MPTs, mentalizing, emotion dysregulation, and PR distress relate to psychopathological symptoms in the context of the COVID-19 pandemic. The current study comprised an online-based and cross-sectional study design, presenting data from the PACE study (see study protocol; Volkert et al., 2021)^1^. Our hypotheses were as follows:

1. PR-distress is (a) positively associated with current symptoms of depression and anxiety, (b) positively associated with MPTs, (c) negatively associated with mentalizing, and (d) positively associated with emotion dysregulation.
2. All forms of childhood trauma are positively associated with MPTs.
3. MPTs are positively associated with symptoms of depression and anxiety.
4. The relationship between MPTs and symptoms of depression and anxiety is partially mediated by (a) mentalizing and (b) emotion dysregulation. Specifically, there is a parallel mediation of the association between MPTs and symptoms of depression and anxiety via mentalizing and emotion dysregulation.
5. There is a correlative relationship between the two mediators mentalizing and emotion dysregulation.

## 2 Materials and methods

### 2.1 Procedure and participants

The convenience sample was recruited over the course of 6 months (August 2020–February 2021) via various social media channels, mails, or flyers and covered German speaking countries. After extensive data cleaning, 182 individuals were removed from the original dataset (detailed information on the data cleaning process can be found in section 2.3., Statistical analyses), ultimately resulting in a final *N* = 6,451 (mean age = 44.1; *SD* = 11.8; 69.1% female, 30.3% male, 0.01% diverse).

The PACE study was approved by the responsible ethics committee. All participants were advised of the study’s aims and provided informed consent before completing the survey.

### 2.2 Measures

#### 2.2.1 Sociodemographic data

Sociodemographic data collection included information on age, gender, educational degree, current employment, family status, and relevant employment-changes due to the COVID-19 pandemic (see Appendix A).

#### 2.2.2 Childhood trauma

The Childhood Trauma Questionnaire Short Form (CTQ-SF; Bernstein et al., 2003) was used for the retrospective assessment of traumatic childhood experiences. The 28-item inventory provides the assessment of the presence and severity of five childhood trauma categories (*emotional*, *physical*, and *sexual abuse* as well as *emotional* and *physical neglect*). Except for *physical neglect* [present study: *α* = 0.52; German validation studies (Dudeck et al., 2015, Klinitzke et al., 2012): 0.55 ≥ *α* ≥ 0.61] the examination of internal consistencies showed good values for four categories [present study: α ≥ 0.85; German validation studies (Dudeck et al., 2015; Klinitzke et al., 2012): α ≥ 0.80].

#### 2.2.3 Maladaptive personality traits

The Personality Inventory for DSM-5 and ICD-11 Brief Form (PID5BF+; Kerber et al., 2022) is a 34-item instrument designed to assess MPTs. The items are assigned to six domains (*negative affectivity, antagonism, disinhibition, detachment, psychoticism,* and *anankastia*), higher values indicating a higher expression of the pathological traits equivalent to a lower level of personality functioning. Reliabilities of the domains in a nonclinical German sample were good (0.83 ≤ *α* ≤ 0.88) except for *anankastia* (*α* = 0.53). Convergent validity between the PID5BF+ and the original PID-5 trait-domain scales showed sufficient results (0.87 < *r* < 0.94; Kerber et al., 2022). In the present study, with the exception of *anankastia* (*α* = 0.59) and *antagonism* (*α* = 0.67) all subscales showed acceptable internal consistency (0.71 < α < 0.78).

#### 2.2.4 Mentalizing

The Mentalization Scale (MentS: Dimitrijević et al., 2018) is a self-report instrument to assess mentalizing. It consists of 28 items, which in turn are assigned to three subscales [*self-related mentalization* (MentS-S), *other-related mentalization* (MentS-O), and *motivation to mentalize* (MentS-M)]. Within the validation study, internal consistencies of the subscales were acceptable in almost each case (0.74 ≤ *α* ≤ 0.79; Dimitrijević et al., 2018) except for the MentS-M scale in the clinical sample (*α* = 0.60). A study examining the construct validity in a German-speaking but mixed-psychiatric sample displayed almost comparable internal consistencies of the subscales of the MentS (Richter et al., 2021). In the present study, an adapted version of the MentS was used (for further informarion see section 2.4, Preparatory analyses): MentS-O (*α* = 0.78) and MentS-M (*α* = 0.87) showed acceptable/good internal consistencies, whereas MentS-S displayed a debatable Cronbach’s alpha of *α* = 0.50.

#### 2.2.5 Emotion dysregulation

The Difficulties in Emotion Regulation Scale – Short Form (DERS-SF; German version: Ehring et al., 2008) was used to assess emotion dysregulation. Using 18 items, it allows the assessment of six different facets of emotion dysregulation (*nonacceptance of emotional responses, difficulties engaging in goal-directed behavior, impulse control difficulties, lack of emotional awareness, limited access to emotion regulation strategies, and lack of emotional clarity*). Examined in a US-American college sample, Cronbach’s alpha coefficient for each subscale ranged from 0.78 ≤ *α* ≤ 0.91 (Kaufman et al., 2016). The DERS-SF is well-validated and widely used in studies investigating adult samples (Hallion et al., 2018). In the present study, an adapted DERS-SF version was applied due to an insufficient internal consistency of the subscale *lack of emotional awareness* (*α* = 0.24; see section 2.4, Preparatory analyses). The subscales of the adapted DERS-SF version displayed good values (0.75 ≤ α ≤ 0.89).

#### 2.2.6 Psychopathological symptoms

The Patient Health Questionnaire Anxiety-Depression Scale (PHQ-ADS; Kroenke et al., 2016) was used as an instrument to quantify psychological distress in terms of anxiety and depression symptomatology. The questionnaire is composed of a depression module (PHQ-9; Löwe et al., 2004) extracted from the Patient Health Questionnaire (PHQ-D; Gräfe et al., 2004) and an anxiety module (GAD-7; Löwe et al., 2008), consisting of 16 items. The symptoms queried are based on the DSM-5 criteria for assigning a depression or an anxiety disorder. Kroenke et al. (2016) tested both the individual scales of the GAD-7 and PHQ-9 and their combination in form of the PHQ-ADS in an US-American sample. Every score showed good internal consistencies (0.80 ≤ α ≤ 0.90) in each of three tested samples, which was confirmed in the present study (α_depression_ = 0.87; α_anxiety_ = 0.90).

#### 2.2.7 Pandemic-related distress

PR distress was assessed by using a Pandemic-related Adversity Score (PrAS) consisting of a series of self-composed items^2^. The 18 items were initially combined into three subscores using content analysis (*distress due to contact restrictions*, *distress due to a change in lifestyle in the context of the pandemic*, *perceived lack of medical and/or psychotherapeutic care*; the exact item formulation can be obtained from Appendix B). Subsequently, a confirmatory factor analysis (CFA) was conducted to test both whether the individual items could be grouped into subscales and whether the subscales loaded univariately on a common overall factor.

### 2.3 Statistical analyses

Statistical analyses were performed using RStudio (R-Core-Team, 2020) and the lavaan package for the structural equation model (SEM) analyses (Rosseel, 2012). Data cleaning consisted of multi-part analyses. After an initial consideration of participation requirements, data were examined for three different types of response anomalies. First, careless responders, characterized by an excessively fast response time or continuously selecting the same response category despite negatively coded control items, were removed from the data set (45 participants). Second, low quality responders with a particularly high percentage of missings [>25%; Collins et al. (2001); 24 participants] or completely missing data of the main constructs (15 participants) were excluded. Finally, a multivariate outlier analysis was performed, which led to the exclusion of 98 participants. Data cleaning resulted in a final sample of *N* = 6,451. Missing data were handled using multiple imputation. A CFA was conducted to assess the factor structure of the PrAS (results can be found in section 2.4, Preparatory analyses). In order to test our hypotheses, one overall SEM in form of a double mediation with two consecutive predictors was calculated. The double mediation allows the two possible meditating processes to be examined simultaneously and in an all-encompassing model. To evaluate both CFA and SEM, three model fit indices were used as indicators of the model quality. The *Goodness of Fit Index* (GFI) as well as the *Comparative Fit Index* (CFI) both indicate an acceptable model fit with values ≥0.90 and a good model fit with values above 0.95. The third index, *Root Mean Square Error of Approximation* (RMSEA), shows an acceptable model fit with values ≤0.08 and values under 0.05 indicating a good model fit (Bollen and Long, 1993). After the SEM was evaluated regarding model fit, it was expanded by including control parameters (variables: age, gender, COVID-19-health-report of own person, COVID-19-health-report of close others), omitting variables resulting in poor fits and allowing intercorrelations (Saris et al., 1987).

### 2.4 Preparatory analyses

**Figure 1:**
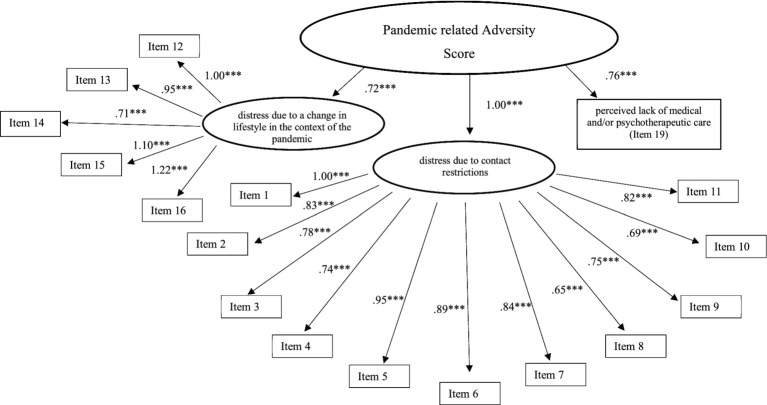
Factor and item loadings of the PrAS. ***p < 0.001.

First, the measurement models of the individual constructs were examined. After allowing theory-compliant intercorrelations, almost all measurement models provided a sufficient fit (CFI ≥ 0.92; GFI ≥ 0.91; RMSEA ≤0.061)^3^. Only the measurement model of the MentS (CFI = 0.85; GFI = 0.92; RMSEA = 0.056) and DERS-SF (CFI = 0.94; GFI = 0.96; RMSEA = 0.113) did not show an acceptable fit to the data. Regarding DERS-SF, this was due to insufficient loading of the subscale score of *lack of emotional awareness* onto the total factor (*b* = 0.15). Since this subscale had already shown low internal reliability (*α* = 0.24), we decided to exclude it from further analyses. Subsequently, the resulting measurement model improved (CFI = 1.00; GFI = 1.00; RMSEA = 0.021). The insufficient model indices of the MentS were due to the low reliability of the MentS-S scale (α = 0.28). Three out of eight items showed a negative loading on the MentS-S scale. The specific wording of the affected items was: (1) “When I get upset, I am not sure whether I am sad, afraid, or angry.” (2) “Often I cannot explain, even to myself, why I did something.” (3) “I do not want to find out something about myself that I will not like.” We ultimately decided to exclude these items and to calculate the MentS-S subscale score from the five remaining items. Hence, Cronbach’s alpha of MentS-S scale increased to *α* = 0.50. Furthermore, a CFA of the content-analytically developed PrAS was performed. The resulting model including the corresponding factor and item loadings can be seen in Figure 1. Overall, the model fit indices of the final SEM indicated a good fit (CFI = 0.90, GFI = 0.87, RMSEA = 0.044; *χ*^2^ = 20010.33, df = 1744, *p* < 0.001). Given the large sample size, the *χ*^2^-value, did not indicate a good fit of the model.

## 3 Results

### 3.1 Descriptive statistics

Sociodemographic data of the final sample as well as pandemic-related descriptive data are depicted in Appendix A. The intercorrelations between variables can be found in Table 1. Most constructs showed intercorrelations as expected. However, contrary to our expectations, the association between mentalizing and anxiety as well as PR distress was positive. Based on the cut-off values for the CTQ subscales according to Hauser et al. (2011), the prevalence rates of traumatic experiences with at least moderate severity were: emotional abuse: 8.22%; physical abuse: 18.74%; sexual abuse: 11.39%; emotional neglect: 51.34%; physical neglect: 1.44%.

**Table 1:** Descriptive values and inter-construct correlations.

Variables	*M*	*SD*	1.	2.	3.	4.	5.	6.	7.
CTQ-SF	38.46	13.38	(0.82)						
DERS-SF	12.49	3.60	0.24^***^	(0.80)					
MentS	102.80	11.73	0.02	−0.27^***^	(0.65)				
PHQ-9	10.16	5.91	0.17^***^	0.41^***^	0.00	(0.87)			
GAD-7	8.10	5.37	0.20^***^	0.46^***^	0.06^***^	0.68^***^	(0.90)		
PID5BF+	4.84	2.06	0.26^***^	0.65^***^	−0.24^***^	0.30^***^	0.36^***^	(0.75)	
PrAS	0.00^a^	.57^a^	0.13^***^	0.19^***^	0.08^***^	0.62^***^	0.51^***^	0.11^***^	(0.52)

Cronbach’s alpha are presented in the main diagonal. *M* = arithmetic mean. SD = standard deviation. CTQ = Childhood Trauma Questionnaire Short Form; DERS-SF = Difficulties in Emotion Regulation Scale Short Form; MentS = Mentalizing Scale; PHQ-9 = Patient Health Questionnaire 9; GAD-7 = General Anxiety Disorder Scale 7; PID5BF+ = Personality Inventory for DSM-5 and ICD-11 Brief Form; PrAS = Pandemic-Related Adversity Score (based on self-developed instrument). ^a^z-standardized values.

^***^p < 0.001.

### 3.2 Hypotheses testing

**Figure 2:**
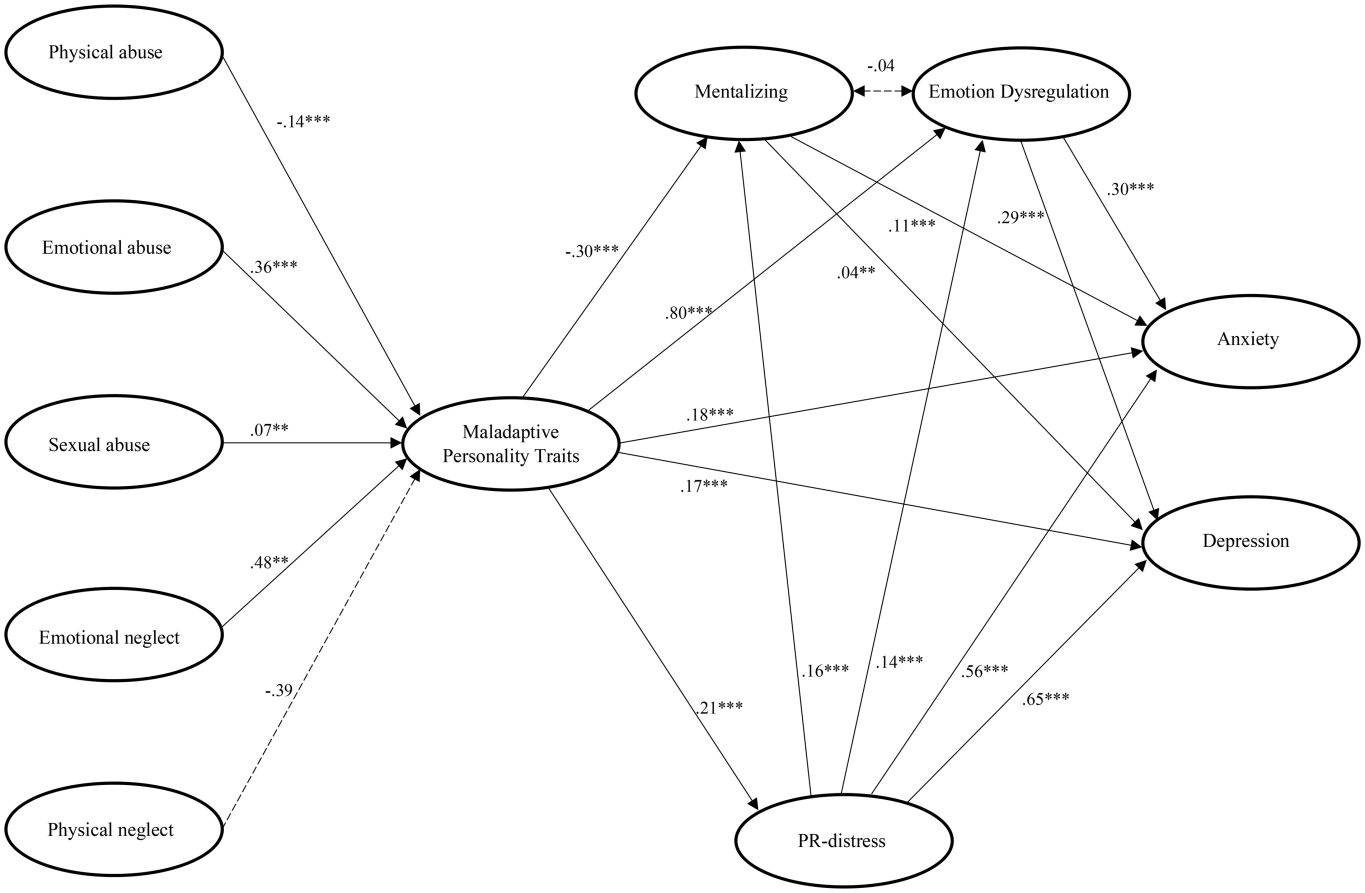
Final SEM. ***p < 0.001, **p < 0.01, *p < 0.05. Measurement models and control variables (age, sex) are not shown for readability. Further information can be requested from the authors.

A comprehensive SEM with associated regression parameters is shown in Figure 2. The results of the individual hypotheses tests are described in detail in the following:

As expected in Hypothesis 1a, both, depression and anxiety, showed a significant, positive association with PR distress (β_anxiety_ = 0.56, β_depression_ = 0.65). According to Hypothesis 1b, a negative relationship between PR distress and MPTs (β = 0.21) was found. However, contrary to Hypothesis 1c, a significant, positive relationship was found between PR distress and mentalizing (β = 0.16). Lastly, Hypothesis 1d was supported showing in that PR distress was positively associated with emotion dysregulation (β = 0.14).

Hypothesis 2 was partly supported by our data. Four out of five CTQ-SF-subscales showed significant associations with MPTs. *Emotional* (β = 0.36) and *sexual abuse* (β = 0.07), and *emotional neglect* (β = 0.48) showed a positive relationship with MPTs consistent with our hypothesis. However, *physical abuse* showed a significant negative association with MPTs (β = −0.14). Likewise, contrary to our expectations, the subscale *physical neglect* revealed no significant association to MPTs (β = −0.39, *p* = 0.094).

Our data are also in line with Hypothesis 3 in that higher values on the MPT dimensions are associated with a greater degree of depressive and anxiety symptoms during the pandemic (β_anxiety_ = 0.18, β_depression_ = 0.17).

The analysis of the SEM revealed a partial mediation via mentalizing for MPTs (see Table 2 for the corresponding mediation coefficients), which was, however, not in the direction expected in Hypothesis 4a. MPTs were indeed significantly associated with lower mentalizing ability (β = −0.30). However, the regression coefficients of mentalizing on anxiety as well as depression (β_anxiety_ = 0.11, β_depression_ = 0.04) suggest that better mentalizing was associated with higher levels of anxiety and depression, despite of the small size of the parameters. Thus, while partial mediation is in line with the hypothesis, it is not in the expected direction between mentalizing and depression or anxiety.

**Table 2:** Regression coefficients of the mediational analyses.

Mediation pathway	Coefficient
Mediation via emotion dysregulation	
Maladaptive Personality Traits ➔ Emotion Dysregulation ➔ Anxiety	0.24***
Maladaptive Personality Traits ➔ Emotion Dysregulation ➔ Depression	0.23***
Mediation via mentalizing	
Maladaptive Personality Traits ➔ Mentalizing ➔ Anxiety	−0.03***
Maladaptive Personality Traits ➔ Mentalizing ➔ Depression	0.01**

^***^p < 0.001, ^**^p < 0.01, ^*^p < 0.05.

In accordance with Hypothesis 4b, the SEM provided evidence that the relationship between MPTs and both depression and anxiety was partially mediated by the degree of emotion dysregulation. In other words, MPTs were significantly positively associated with lower emotion dysregulation (β = 0.80), which in turn had a significant positive effect on anxiety (β = 0.30) and depression (β = 0.29).

According to hypothesis 5, the two mediators, mentalizing and emotion dysregulation were expected to be associated with each other. However, the final SEM revealed no significant association (*r* = −0.04, *p* = 0.098) between the two mediators, disproving Hypothesis 5. Accordingly, the final model shows two distinct parallel mediations.

## 4 Discussion

This study investigated the associations of childhood trauma, MPTs, mentalizing, and emotion dysregulation as transdiagnostic factors and underlying mechanisms relevant to psychopathological symptoms during the COVID-19 pandemic.

First, we found a significant, positive association between PR distress and depressive and anxiety symptoms. This expands findings from previous studies, which found increases in psychological distress and psychopathological symptoms during the pandemic using established and validated measures of psychopathology in general (e.g., Robinson et al., 2022), which did not allow determining whether psychopathological symptoms were due to the pandemic or other factors. By utilizing a measure of PR distress, our study contributes to delineating the extent distress and symptoms were attributed to the pandemic, whereby subjectively perceived PR distress leads to an increase in psychopathological symptoms. Considering the subjective nature of the self-report questionnaire for the assessment of PR-distress and the cross-sectional study design, it remains unclear whether this measure distinguishes between subjective and objective distress and whether the experience of more PR-distress is attributable to preexisting symptoms of depression and anxiety.

Second, our analysis revealed a positive relationship between PR distress and MPTs, indicating that individuals with MPTs were at higher risk for experiencing more PR distress. Our finding supports the few previous empirical results showing that MPTs were associated with psychological distress during the pandemic (e.g., Sica et al., 2021). Accordingly, this finding supports the assumption that individuals with MPTs are more vulnerable to experiencing the pandemic as more stressful.

Third, our hypothesis on a negative relationship between PR distress and mentalizing was not confirmed, in contrast, we found a significant, positive relationship. Similarly, a significant, positive association between anxiety and mentalizing was found. This contradicts the theoretical assumptions on the association between anxiety and mentalizing as well as the existing albeit limited literature showing that mentalizing was negatively associated with perceived stress in adolescents during the pandemic (Locati et al., 2023). One possible interpretation of our finding is related to the mentalizing measure utilized in our study, the MentS. Higher scores on the MentS may actually reflect aspects of *hypermentalizing*, which refers to a form of excessive elaboration about mental states in oneself and others. This type of reflection, however, is not authentically connected to real life events and considered a specific type of the prementalizing pretend mode (Luyten et al., 2020). Thus, rather than reflecting increased mentalizing capacity, higher scores on some MentS items (e.g., “*I often think about other people and their behavior*”) may be considered an expression of impaired mentalizing. Furthermore, relating these findings to clinical presentation and theory, in a highly anxious state an individual may pseudo-mentalize as a kind of coping and protective mechanism to regulate anxiety (in a maladaptive manner). Similarly, constant worrying, which can also be considered a type of maladaptive coping in dealing with anxiety and can reflect hypermentalizing, could lead to higher scoring on particular items of the MentS.

Fourth, our hypothesis on a positive association of PR distress with emotion dysregulation was supported. This is in line with previous studies demonstrating that emotion regulation difficulties are related to elevated psychological distress (Siegel and Lahav, 2021). So far, most empirical findings focused on the assessment of emotion regulation strategies instead of broad facets of emotion dysregulation beyond processing and regulating emotions. Considering that the DERS captures a broader concept of emotion dysregulation, our finding expands the literature on pandemic-related associations with emotion dysregulation. Consequently, emotion dysregulation could be understood as a risk factor for PR distress.

Fifth, our hypothesis stating that all types of childhood trauma would show a positive association with MPTs, was partially confirmed. Four of five CTQ-subscales (*emotional, physical, and sexual abuse* as well as *emotional neglect*) showed significant associations with MPTs. This finding is in line with a recent review indicating significant associations between sexual, physical, and emotional abuse with MPTs (Back et al., 2021). However, the subscale *physical neglect* revealed no significant association with MPTs. A possible explanation for this finding may be related to the low internal consistency of this subscale in our study, which is in line with previous findings across different samples (incarcerated, clinical, and community samples; e.g., Aizpurua et al., 2024; Dudeck et al., 2015) and may indicate weaknesses in the original construction of this subscale. For instance, the item “*got taken care of*” (reverse scoring) may be interpreted to reflect both emotional and physical neglect.

Sixth, hypothesis 3 was confirmed, whereby MPTs were positively associated with more severe psychopathological symptoms during the pandemic. This finding is in line with the existing literature showing that internalizing personality traits (i.e., detachment, negative affect, psychoticism) were significantly associated with depressive and anxiety symptoms (Mazza et al., 2020) during the pandemic. Consistent with hypothesis 4, we also found that the association between MPTs and psychopathological symptoms was partly mediated by mentalizing and emotion dysregulation. Thereby MPTs were significantly positively associated with emotion dysregulation, which in turn had a significant positive effect on anxiety and depression. This is in line with previous studies showing that emotion dysregulation serves as a mediator in the relationship between MPTs and psychopathology (e.g., Abdi and Pak, 2019; Herr et al., 2013). Our finding further supports the role of emotion dysregulation as a partial mediator between MPTs and psychopathology in a nonclinical sample, making it a promising key to target in prevention of mental health problems under distress. Surprisingly, we found that higher scores in mentalizing were associated with more anxiety and more depressive symptoms during the pandemic, contrary to our hypothesis. This result may also be attributable to the MentS capturing aspects of hyper-or non-mentalizing, which has been shown to be associated with psychopathology (McLaren et al., 2022). Further external validation of the MentS would be necessary to investigate this.

Lastly, hypothesis 5 was not supported as the final SEM revealed a non-significant correlation between the two mediators mentalizing and emotion dysregulation: the final model showed two significant parallel mediations with uncorrelated mediators. Hence, emotion dysregulation as well as mentalizing both seem to be relevant for an individual’s psychological reaction to the pandemic but in distinct ways. On a conceptual level, the constructs differ substantially: while the construct emotion dysregulation captures the incapacity to flexibly process, perceive, and understand emotions, the ability to mentalize is captured by the different aspects of reflecting *self-related mentalizing, other-related mentalizing*, and *motivation to mentalize*. Accordingly, both constructs seem to differ greatly. Our finding contradicts previous models proposing correlative associations between these two constructs (e.g., Ciccarelli et al., 2021), which may be related to higher scores on our mentalizing measure potentially reflecting hypermentalizing.

To the best of our knowledge, this is the first study investigating the associations of childhood trauma, MPTs, mentalizing, and emotion dysregulation as transdiagnostic factors and underlying mechanisms relevant to psychopathological symptoms during the COVID-19 pandemic. Further, by developing and including a measure for the assessment of PR distress, we aimed to differentially assess and thereby disentangle the effects of self-reported distress attributed to the pandemic and psychopathological symptoms, in particular depression and anxiety. A major strength of this study pertains to a large sample size of *N* = 6,451 participants as well as the subsequent extensive data cleaning procedure. Our findings provide information on key targets for pre-and intervention methods that could be used to prevent or treat multiple types of psychopathologies during the pandemic.

The following limitations of our study need to be considered: The cross-sectional study design only shows correlational associations and does not allow any causal interpretation of the findings. As this study started during the pandemic, pre-pandemic data of participants was lacking. Without pre-pandemic scores, we cannot conclude about changes due to the pandemic and their predictors. Furthermore, while our results support an association between PR distress and psychopathological symptoms, we cannot assume a direction of effects. It seems likewise plausible that more depressed and anxious individuals experience more PR distress. Further, the self-report questionnaires utilized are retrospective, subjective in nature and therefore susceptible to recall bias, particularly, the assessment of childhood trauma. However, it was shown that subjective memories, including retrospective designs, of childhood maltreatment have clinical relevance (Danese and Widom, 2020). Further, the MentS revealed psychometric problems (low reliabilities of one subscale) which may be reflected in our findings. Research has demonstrated that low reliabilities of questionnaires can result in adverse effects on the effect size, power of hypothesis tests, and replicability of results across different statistical methods (Cleary et al., 1970; Humphreys and Drasgow, 1989; LeBel and Paunonen, 2011) While the precise impact of the MentS’s poor psychometric properties on our results remains uncertain, the results must be interpreted with this limitation in mind. Furthermore, it is crucial to acknowledge that the PrAS was developed within the context of this study and, as a result, could not be externally validated. Future research on the validity of the PrAS is warranted. Although our study presents a convincing sample size of *N* = 6,451, the sample might be subject to biases of a population primarily recruited online that were easily reached. A self-selection bias may be considered with regard to a higher education level and female ratio compared to Germany‘s general population sample. Regarding the educational level, a major amount of our sample holds a university degree (75%), in contrast to Germany’s general population sample with 33.5% (Federal Statistical Office, 2020). Regarding the gender ratio, our study’s sample is mostly female with 75%, whereas the gender ratio of Germany’s population sample is balanced (Federal Statistical Office, 2024). Despite this, it should be emphasized that an online-based study implies the possibility of reaching individuals beyond regional limitations. Lastly, the significant *χ*^2^ could either be related to an insufficient fit of the model or to the large sample size. Previous literature has shown that the *χ*^2^-test for sample-sizes greater than 200 will always be significant due to a high *T*-value (Babyak and Green, 2010). Correspondingly, only small discrepancies between the implied and observed model will yield significance. Therefore, given our large sample size, it is recommended to consider other model-fit indices, such as CFI, GFI, and RMSEA, when evaluating the model which has been done in the present study.

## 5 Conclusion

Taken together, our findings contribute to an overarching understanding of transdiagnostic mechanisms associated with psychopathological symptoms under PR distress. Several implications can be derived: (1) based on our findings mentalizing and emotion dysregulation may be considered as key psychological capacities impacting an individual’s ability to adjust to the pandemic or other crises events. The provision of specific pre−/intervention methods that target emotion dysregulation and mentalizing could help vulnerable individuals – particularly those with childhood trauma and MPTs−to protect against or alleviate detrimental effects of PR distress on their mental health. (2) Future longitudinal research on the fluctuation or stability of MPTs, mentalizing, and emotion dysregulation and their associations as well as effects on distress and psychopathological symptoms over time during the pandemic or other crises events is needed. Moreover, using intensive longitudinal assessment methods (e.g., ecological momentary assessment) it would be possible to investigate contextual dynamics of MPTs, mentalizing, and emotion dysregulation to uncover when these underlying mechanisms are relevant and how. (3) Furthermore, future studies should focus on intraindividual differences of those underlying mechanisms for in-depth exploration on the distinction between mentalizing and emotion dysregulation. At the individual level different profiles might be possible, e.g., for one individual, *mentalizing the self* could be a key mediator between MPTs and psychopathology whereas for another individual it is *emotion dysregulation of specific negative emotions*.

## Data Availability

The raw data supporting the conclusions of this article will be made available upon reasonable request by the authors, without undue reservation.

## Footnotes

^1^Please note that *The Standardized Assessment of Severity of Personality Disorder* (SASPD), a screening tool for personality functioning, was excluded from further statistical analysis due to psychometric inconsistencies. Accordingly, we formulated the hypotheses in relation to maladaptive personality traits.

^2^The original item pool additionally included items on physical health during the pandemic. Due to an insufficient factor loading of the psychosocial items on a common factor, they were excluded from the PrAS score formation. The original items can be obtained from the authors.

^3^More detailed information on the measurement models can be obtained from the authors.
