# Supplementary material for: Transdiagnostic mechanisms of mental health during the COVID-19 pandemic: associations of childhood trauma, maladaptive personality traits, emotion regulation, mentalizing, and pandemic-related distress
Source: Front Psychol. 2024 Dec 18;15:1427469. doi: 10.3389/fpsyg.2024.1427469 (PMC11688181; doi:10.3389/fpsyg.2024.1427469)
Supplement: Supplementary file 1 [file Table_1.DOCX]

**Appendix A**

Sociodemographic characteristics (*N* = 6,451)

| Sociodemographic variable | *n* (%) | | |
| --- | --- | --- | --- |
| Highest educational attainment |  | | |
| Finished school without a diploma | 9 | (0.14) | |
| Currently in school | 12 | (0.19) | |
| Middle school diploma | 63 | (0.98) | |
| Completed apprenticeship | 679 | (10.53) | |
| High school diploma or similar | 2534 | (39.28) | |
| College/university degree | 2916 | (45.21) | |
| Other | 237 | | (3.67) |
| Current employment |  | | |
| Student in school | 13 | | (0.20) |
| In training | 79 | | (1.23) |
| Student in university/college | 572 | | (8.87) |
| Employee | 3572 | | (54.70) |
| Civil Servant | 509 | | (7.89) |
| Self-employed | 888 | | (13.77) |
| Unemployed/Job-seeking | 199 | | (3.09) |
| Other | 661 | | (10.25) |
| Change in employment situation due to the pandemic |  | | |
| Reduction (short-time work) | 753 | | (11.67) |
| Homeoffice | 1949 | | (30.21) |
| Total discontinuation of work | 256 | | (3.97) |
| Other changes | 1788 | | (27.72) |
| No changes | 2182 | | (33.28) |
| Current monthly income |  | | |
| 0€ to 1000€ | 1430 | | (22.58) |
| 1000€ to 2000€ | 1736 | | (27.42) |
| 2000€ to 3000€ | 1646 | | (25.99) |
| 3000€ to 5000€ | 1158 | | (18.29) |
| 5000€ or more | 362 | | (5.72) |
| Reduction of income due to the pandemic |  | | |
| Yes | 1589 | | (24.70) |
| No | 4843 | | (75.30) |
| In a relationship | 4646 | | (72.02) |
| Having children | 3714 | | (55.99) |

*Note*. *n* = number of participants
