# Supplementary material for: Transdiagnostic mechanisms of mental health during the COVID-19 pandemic: associations of childhood trauma, maladaptive personality traits, emotion regulation, mentalizing, and pandemic-related distress
Source: Front Psychol. 2024 Dec 18;15:1427469. doi: 10.3389/fpsyg.2024.1427469 (PMC11688181; doi:10.3389/fpsyg.2024.1427469)
Supplement: Supplementary file 2 [file Table_2.DOCX]

**Appendix B**

Means (SD) and Frequencies of the Pandemic-related Adversity Scale (PrAS) Items (*N* = 6,451)

|  |  |  | *n* (%*)* of responses per response category | | | | | | | | | | | | | | | |
| --- | --- | --- | --- | --- | --- | --- | --- | --- | --- | --- | --- | --- | --- | --- | --- | --- | --- | --- |
| Score | No. | Item | *M (SD)* | 0 | | 1 | | | 2 | | | | | 3 | 4 | | | Missings |
| Distress due to contact restrictions ^a^ | 1 | Do you feel the restrictions imposed by the contact regulations are a burden? | 4.01  (1.24) | 340 (5.27) | | 664  (10.29) | | | 855  (13.25) | | | | | 1286  (19.93) | 3299  (51.14) | | | 7  (0.11) |
|  |  | I feel or have felt the restrictions imposed by the contact regulations to be a burden because… |  |  | |  | | |  | | | | |  |  | | |  |
|  | 2 | ... I feel constricted at home. | 1.76  (1.60) | 2333  (36.16) | | 804  (12.46) | | | 744  (11.53) | | | | | 1184  (18.35) | 1380  (21.39) | | | 6 (0.09) |
|  | 3 | ... at the moment I have no access to important contact points (offices/counseling centers/therapy/doctors/midwives/courses/others). | 2.09  (1.52) | 1142  (17.70) | | 721  (11.18) | | | 795  (12.32) | | | | | 937  (14.52) | 1240  (19.22) | | | 1616 ^b^  (25.05) |
|  | 4 | ...I lack social contact (e.g., with friends, parents). | 3.08  (1.25) | 472  (7.32) | | 799  (12.39) | | | 1319  (20.45) | | | | | 3477  (53.90) | 379  (5.88) | | | 0 (0.00) |
|  | 5 | ... my freedom is restricted. | 2.70  (1.6) | 1179  (18.28) | | 625  (9.69) | | | 570  (8.84) | | | | | 668  (10.35) | 3399  (52.69) | | | 10  (0.16) |
|  | 6 | ... I feel left alone. | 1.77  (1.60) | 2309  (35.79) | | 774  (12.00) | | | 922  (14.29) | | | | | 965  (14.96) | 1472  (22.82) | | | 9  (0.14) |
|  | 7 | ... I can no longer pursue my hobbies. | 2.54  (1.52) | 1170  (18.14) | | 574  (8.90) | | | 920  (14.26) | | | | | 1175  (18.21) | 2608  (40.43) | | | 4  (0.06) |
|  | 8 | I experience social contact via media (e.g., telephoning, Skype, WhatsApp) as positive and supportive. * | 1.92  (1.33) | 1196  (18.54) | | 1335  (20.69) | | | | 1725  (26.74) | | | | 1163  (18.03) | 1027  (15.92) | | | 5  (0.08) |
|  | 9 | Social contact via media puts a strain on me. | 1.66  (1.39) | 1875  (29.07) | | 1238  (19.19) | | | | 1329  (20.60) | | | | 1203  (18.65) | 794  (12.31) | | | 12  (0.19) |
|  | 10 | I feel relieved by the reduced social contact. * | 3.09  (1.72) | 247  (3.83) | | 559  (8.67) | | | | 1092  (16.93) | | | | 1124  (17.42) | 3423  (53.06) | | | 2 (0.10) |
|  |  |  |  |  | |  | | | |  | | | |  |  | | |  |
|  |  |  | *n* (%*)* of responses per response category | | | | | | | | | | | | | | | |
| Score | No. | Item | *M (SD)* | 0 | | 1 | | | 2 | | | | | 3 | 4 | | | Missings |
|  | 11 | I think I am up to the challenges posed by the Corona pandemic. * | 1.75  (1.24) | 1169  (18.12) | | 1688  (26.17) | | | | 1882  (29.17) | | | | 974  (15.10) | 730  (11.32) | | | 8  (0.12) |
|  |  |  | *n* (%) of responses per response category | | | | | | | | | | | | | | | |
| Score | No. | Item | *M (SD)* | | 0 | | | 1 | | | | 2 | | | 3 | | Missings | |
| Distress due to a change in lifestyle ^c^ |  | What changes do you notice in yourself since the start of the Corona pandemic? In terms of… |  | | | | | | | | | | | | | | | |
|  | 12 | …physical health | 0.99 (1.22) | | 3397 (52.66) | | | 1117  (17.32) | | | | 548  (8.49) | | | 1389  (21.53) | | 0 (0.00) | |
|  | 13 | …physical activity | 1.53 (1.27) | | 2163 (33.35) | | | 949  (14.71) | | | | 1107  (17.16) | | | 2232  (34.60) | | 0 (0.00) | |
|  | 14 | …nutrition | 0.70 (1.09) | | 4346 (67.37) | | | 780  (12.09) | | | | 431  (6.68) | | | 892  (13.83) | | 2  (0.03) | |
|  | 15 | …pursuing hobbies | 1.74 (1.34) | | 2102 (32.58) | | | 468  (7.25) | | | | 787  (12.20) | | | 3083  (47.79) | | 56  (0.87) | |
|  | 16 | …experienced creativity | 1.31 (1.30) | | 2797 (43.36) | | | 793  (12.29) | | | | 894  (13.86) | | | 1966  (30.48) | | 1 (0.02) | |
|  |  |  | *n* (%) of responses per response category | | | | | | | | | | | | | | | |
| Score | No. | Item | Yes | | | | | | | | No | | | | | | | |
| Lack of medical and/or psycho-therapeutic care |  | Are you experiencing a lack of medical or psychotherapeutic care/other care due to the Corona pandemic? |  | | | | | | | |  | | | | | | | |
|  | 17 | … of medical care? | 2041  (31.64) | | | | | | | | 4410  (68.36) | | | | | | | |
|  | 18 | … of psychotherapeutic care? | 1857  (28.79) | | | | | | | | 4594  (71.21) | | | | | | | |
|  |  |  |  | | | | *n* (%) of responses per response category | | | | | | | | | | | |
| Score | No. | Item | *M (SD)* | | | | 0 | | | | | | 1 | | | 2 | | |
|  | 19 | Lack of medical and therapeutic care total ^d^ | 0.60  (0.74) | | | | 3554  (55.09) | | | | | 1896  (29.39) | | | | 1001  (15.52) | | |

*Note*. *n* = number of participants, *M* = arithmetic mean. *SD* = standard deviation

^a^ response categories ranging from “not at all” to “very much”. ^b^ High proportion of missings, as "does not apply" was recoded to missing. ^c^ recoded answer option from "unchanged/better" to "worse. ^d^ metric sum score of items 17 and 18. * negatively coded item, has been recoded.
